# Variation within Variation: Comparison of 24-h Rhythm in Rodent Infarct Size between Ischemia Reperfusion and Permanent Ligation

**DOI:** 10.3390/ijms18081670

**Published:** 2017-08-01

**Authors:** Bastiaan du Pré, Toon Van Veen, Sandra Crnko, Marc Vos, Janine Deddens, Pieter Doevendans, Linda Van Laake

**Affiliations:** 1 Department of Cardiology, University Medical Center Utrecht, 3508GA Utrecht, The Netherlands; b.c.dupre@umcutrecht.nl (B.P.); j.c.deddens@umcutrecht.nl (J.D.); p.doevendans@umcutrecht.nl (P.D.); 2 Department of Medical Physiology, University Medical Center Utrecht, 3508GA Utrecht, The Netherlands; a.a.b.vanveen@umcutrecht.nl (T.V.V.); m.a.vos@umcutrecht.nl (M.V.); 3 Regenerative Medicine Center, University Medical Center Utrecht, 3508GA Utrecht, The Netherlands; s.crnko@umcutrecht.nl

**Keywords:** myocardial infarction, circadian, 24-h, rhythm, permanent ligation, reperfusion, reperfusion damage, infarct

## Abstract

The detrimental effects of myocardial infarction in humans and rodents have a 24-h rhythm. In some human cohorts however, rhythmicity was absent, while the time of maximum damage differs between cohorts. We hypothesized that the type of damage influences the 24-h rhythm in infarct size. Myocardial infarction was induced in 12-week-old C57BL/six mice at four different time-points during the day using either permanent ligation (PL) or 30-min of ischemia followed by reperfusion (IR), with a control group wherein no ligation was applied. Infarct size was measured by echocardiography and histology at a 1-month follow-up. Rhythmicity in infarct size was present in the PL group at the functional and histological level, with maximal damage occurring when the infarct was induced at noon. In the IR group, no circadian rhythm was found. The time of the coronary artery ligation determines the outcome of myocardial infarction. Our data showed that in rodents, the presence of circadian rhythmicity and time of peak infarct size varies between experimental setups.

## 1. Introduction

Twenty-four-hour (diurnal, day/night) rhythms are biorhythms that play an important role in cardiac physiology [1,2]. Cardiovascular parameters, such as blood pressure, heart rate, circulating hormones, and coagulation time all display 24-h oscillations [3]. These rhythms are regulated by two mechanisms: (1) central regulation via the brain, mainly via neurohumoral signaling, and (2) peripheral regulation via molecular circadian clocks present within individual cells [4].

Twenty-four-hour rhythms are not only important in physiology, but also play an important role in cardiovascular disease (CVD). The incidences of sudden cardiac death and myocardial infarction (MI), for example, have a diurnal rhythm, and disruption of a normal day/night rhythm, such as those associated with shift work, leads to an increase in CVD [5,6]. Recently, it was shown that in addition to their role in CVD incidence and pathophysiology, 24-h rhythms are crucial in CVD outcome as well [7,8,9]. In three rodent studies, myocardial infarction induced at four different time-points showed a 24-h rhythm in infarct size [7,8,9].

These animal studies were followed by several clinical studies investigating patients suffering from Segment (ST)-elevated myocardial infarction. Six out of eight studies demonstrated that 24-h oscillations are present in the infarct outcome [10,11,12,13,14,15]. However, two relatively large studies failed to show rhythmicity in echocardiographic parameters and/or cardiac enzymes [16]. In addition, the time of maximum infarct size differed between studies. When symptom onset occurred between midnight and 6 a.m., it was associated with the worst outcome in the majority of studies [10,13,14,15], but two studies had a different peak time (6 a.m. until noon) [11] and two damage peaks, one around 9 a.m. and one around 8 p.m. [12] respectively.

Recently, there has been some debate about what causes the differences between these studies. Several factors, such as differences in ethnic background, medication use, statistical methodology, study size, culprit artery, time of ischemia, comorbidities (diabetes), climate and day–night cycle variations, and outcome measure have been proposed to explain variation between those studies [13,17,18,19,20].

One of the most commonly suggested explanations is the difference in reperfusion damage [18]. In the studies of Ammirati et al. and Mukamal et al., in which no circadian effects in echocardiographic/cardiac enzyme parameters were observed, most patients did not receive primary percutaneous coronary intervention (PCI) [13,21]. The contribution of reperfusion damage to infarct size in these studies is therefore smaller than in the other cohorts. In addition, the data of Durgan et al. and Virag et al. suggest that mitochondrial function mediates the 24-h rhythm in cardioprotection [7,22]. Reperfusion after PCI causes an increase in oxidative stress within the heart, which is specifically harmful for cardiac mitochondria [23].

Based on these findings, we hypothesized that 24-h differences in reperfusion damage are responsible for 24-h rhythmicity in infarct size. To test this hypothesis, we induced myocardial infarction in mice at different time points using either (1) ischemia/reperfusion (IR) or (2) permanent ligation (PL).

## 2. Results

### 2.1. Operations

**Figure 1:**
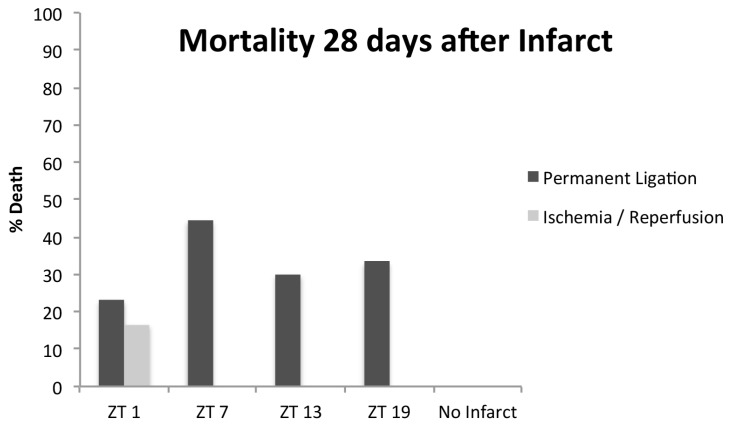
Mortality in the 28 days after the infarct. Thirteen mice died, of which 0 underwent sham, 1 ischemia/reperfusion (IR), and 12 permanent ligations (PLs). In the PL group, mortality was highest in the ZT 7 group, although differences were not statistically significant. ZT, Zeitgeber Time (ZT0 = lights on).

Sixty-five mice were operated. During follow-up, 13 mice (20%; all PL/IR) had died or were euthanized because a humane endpoint was reached. Twelve of the thirteen dead animals had undergone PL, and the majority (11/13) died within the first week after the infarct. Mortality was highest in the animals operated at ZT 7 (the middle of the inactive/resting period), although differences were not statistically significant (Figure 1; *p* = 0.77).

### 2.2. Body and Cardiac Weight

Of the 52 mice that completed the follow-up, 20 were in the PL group, 18 in the IR group, and 14 were controls. Body weight decreased slightly in the 5 days after the infarct (0.6 ± 0.1 g), but increased in the overall 1-month follow-up (27.5 ± 0.1 to 28.3 ± 0.1g). No significant differences in body weight were found between types of ischemia and time-points.

**Figure 2:**
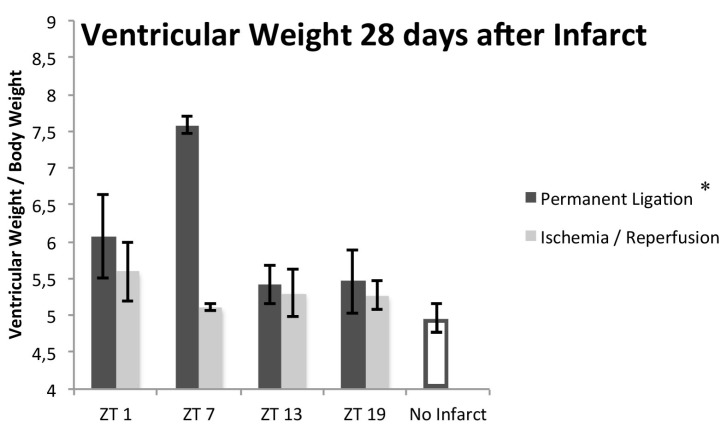
Ventricular weight 28 days after the infarct. Compared to sham controls, PL mice had an increase in biventricular mass that showed a significant 24-h rhythm peaking at ZT7 (the middle of the inactive/resting period). In IR mice, no significant increase or 24-h rhythm was present. *n* = 20, 18, and 14 for PL, IR, and sham respectively; *n* = 3–5 per timepoint. Striped line indicates control value; * Indicates *p* < 0.05 for the presence of 24-h rhythm. ZT: Zeitgeber Time (ZT0 = lights on).

Biventricular weight of mice that had undergone PL was significantly increased compared to the sham group, whereas no significant differences were found between the IR group and the controls (biventricular weight/body weight ratio 6.00 ± 0.27, 5.34 ± 0.53, and 4.96 ± 0.09 mg/g for PL, IR, and sham respectively; *p* = 0.003 and 0.086 for PL and IR vs. sham respectively). In the PL mice, there was a significant difference in biventricular weight (*p* = 0.048, *n* = 20) depending on the time of coronary artery ligation, which was absent in the IR group (Figure 2; *p* = 0.543, *n* = 18).

### 2.3. Echocardiography

**Figure 3:**
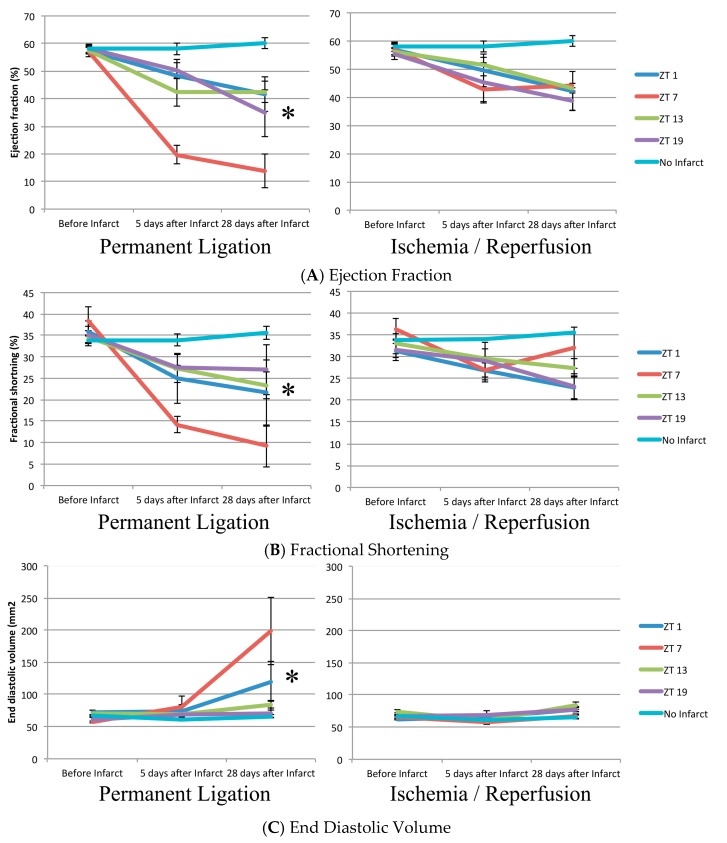
Echocardiographic parameters 5 days before, 5 days after, and 28 days after myocardial infarct. *n* = 20, 18, and 14 for PL, IR, and sham respectively, *n* = 3–5 per timepoint. * Indicates *p* < 0.05 for the presence of 24-h rhythm. ZT, Zeitgeber Time (ZT0 = lights on).

Cardiac function decreased in the PL and IR groups and was significantly worse compared to the sham group 28 days after the infarct (Table 1 and Figure 3). Cosinor analysis revealed that functional impairment varied significantly with a 24-h period based on the timing of coronary artery ligation in the mice receiving PL, with peak damage occurring after coronary artery ligation at ZT7 (the middle of the inactive/resting period; *p* = 0.041, *p* = 0.049, and 0.021 for Left ventricular ejection fraction (EF), fractional shortening (FS), and end diastolic volume (EDV) respectively, *n* = 20). In the IR mice however, no circadian dependence was observed (*p* = 0.598, 0.127, and 0.368 for EF, FS, and EDV respectively, *n* = 18).

### 2.4. Histology

**Figure 4:**
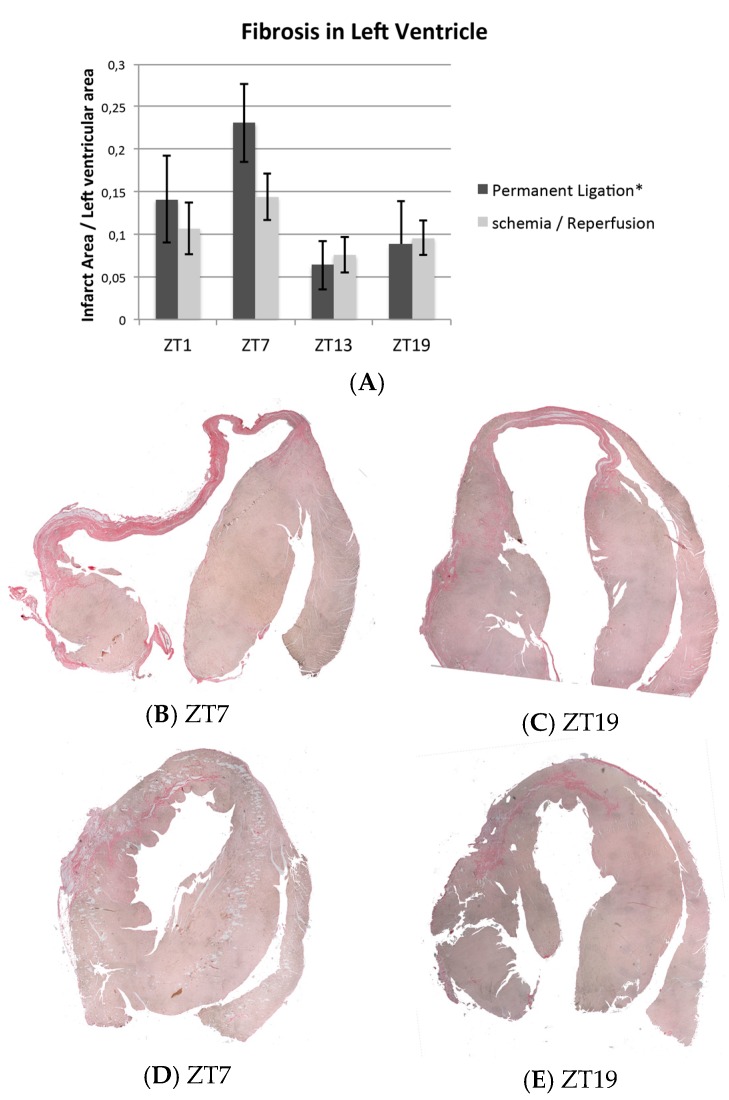
Picosirius-red staining of myocardial infarcts. The pink tissue represents fibrosis. (**A**) Fibrosis quantification; a 24-h rhythm in amount of fibrosis, based on the time of coronary artery ligation was present in PL but not IR. *n* = 20, 18, and 14 for PL, IR, and sham respectively, *n* = 3–5 per time point; (**B**–**C**) representative examples of permanent ligation. Infarcts induced at ZT7 (**B**, middle of inactive/resting period) were larger compared to infarcts induced at ZT19 (**C**, middle of the active period); (**D**–**E**) representative examples of Ischemia/reperfusion. No differences were found between time points. * Indicates *p* < 0.05 for 24-h rhythm. ZT: Zeitgeber Time (ZT0 = lights on).

Fibrosis quantification confirmed the echocardiographic findings and showed a significant 24-h rhythm in the amount of fibrosis based on the time of coronary artery ligation, with peak fibrosis at ZT7 in the PL group (the middle of the inactive/resting period; *p* = 0.024, Figure 4A,B, *n* = 20). In the IR mice, no rhythm was present (*p* = 0.110, *n* = 18; Figure 4A,C).

## 3. Discussion

In the current study, we analyzed 24-h rhythms in outcome of myocardial infarction. For the first time, we directly compared circadian influences in two infarction models that differed with respect to the absence or presence of reperfusion. Thereby, we eliminated all other variables, such as mouse strain, gender, age, anesthesia, or housing conditions, that may have influenced previously published controversial results.

Our histological and functional data show that a diurnal rhythm in infarct size, as measured by the amount of fibrosis, is present in our mouse model of permanent ligation, but not in the ischemia/reperfusion model. These findings contradict our original hypothesis that rhythmicity is caused by reperfusion damage and suggest that differences in rhythmicity (presence of rhythmicity and time of peak damage) found in clinical studies are not solely based on this type of damage.

Our results confirm the findings of Durgan et al., Eckle et al. and Schloss et al. that a 24-h rhythm is present in the outcome of myocardial infarction [7,8,9]. The timing of coronary artery ligation that was associated with peak damage, however, was different between studies (Durgan et al. [7] ZT 6–12, Schloss et al. [9] ZT 13, Eckle et al. [8] ZT 0–6, our data ZT 7). Secondly, Durgan et al. [7] and Eckle et al. [8] found rhythmicity in infarct outcome based on the time of occlusion in IR, whereas we only found a rhythm in PL. There are multiple differences between the studies that might potentially explain these observations. Durgan et al. used a closed-chest infarct model, as opposed to the open-chest model of Eckle et al. [24] and our study [25]. In addition, compared to our IR model, duration of ischemia prior to reperfusion was 50% and 100% longer in the studies of Durgan et al. and Eckle et al. [7,8] respectively, thereby inducing more permanent damage comparable to our permanent ligation model. Furthermore, there were significant differences in mouse strains, echocardiography device (with different baseline echo data), outcome measures, and light/dark schedule. Direct comparison between studies is therefore not possible. Indeed, similar to the clinical studies, these differences illustrate that variability is present in 24-h rhythmicity of infarct outcome.

Our data may have several clinical implications. First, it demonstrates that rhythmicity in infarct outcome is not limited to patients receiving PCI, but may be present in all patients with myocardial infarction. Secondly, it confirms that physiological explanations may underlie variation in preclinical and clinical rhythmicity studies; an absence of rhythmicity or peak differences does not have to be attributed to flaws in study-setup or statistics. Finally, it shows that when studying 24-h rhythms in infarct outcome, physiological parameters such as ethnicity, outcome measures, infarct size, and day–night cycle variations may influence rhythmicity, and should therefore be taken into account. Understanding which parameters influence rhythmicity in infarct outcome may lead to novel ways to minimize infarct damage.

In conclusion, our data confirmed that outcome of myocardial infarction in rodents depends on the time-of-day of the ischemic event and showed that this diurnal rhythm is present in the absence of reperfusion. Furthermore, and similar to clinical studies, our study demonstrated that the presence and peak of 24-h rhythmicity is variable.

## 4. Materials and Methods

### 4.1. Animals

All experiments were carried out in accordance with the Guide for the Care and Use of Laboratory Animals, with prior approval by the Animal Ethical Experimentation Committee, Utrecht University, The Netherlands (2013.II.04.047, 15-05-2015).

Male, wild-type C57BL/6 mice (Charles River, Wilmington, MA, USA) were housed in a 14-h light/10-h dark cycle (Zeitgeber Time 0 (ZT0) = lights on, 14 h later (ZT14) = lights off). Food and water were provided ad-libitum. At 10 weeks of age, the mice were distributed to 3 rooms with different light regimes (light on at 5 a.m., 11 a.m., or 2 p.m., all in a 14-h light/10-h dark cycle) to enable MI operations during working hours.

### 4.2. Myocardial Infarction

Mice were anesthetized with medetomidine 0.5 mg/kg, midazolam 5 mg/kg, and fentanyl 0.05 mg/kg, and MI was induced using an open-chest model as described previously [25]. Three different types of MI were used. (1) PL: permanent left coronary artery ligation; (2) IR: 30 min left coronary artery ligation in which a small tube was placed within the stitch, followed by reperfusion (removal of stitch and tube); (3) controls: incision of a needle and a suture thread, but no ligation. Mice that died or were terminated because a humane endpoint was reached during the operation or follow-up were replaced.

Mice were operated at 4 different time-points (ZT1, ZT7, ZT13, and ZT19). To prevent disturbance of the sleep/wake cycle by the necessary light during the operations, mice were anesthetized before transport to the operation theater. For the mice operated at ZT19, cages were completely covered during transport to the operation theater to ensure darkness was maintained. Subsequently, mice were intubated and positioned with minimal light and after the head of the mouse was completely covered, operation lights were turned on.

### 4.3. Echocardiography

**Figure 5:**
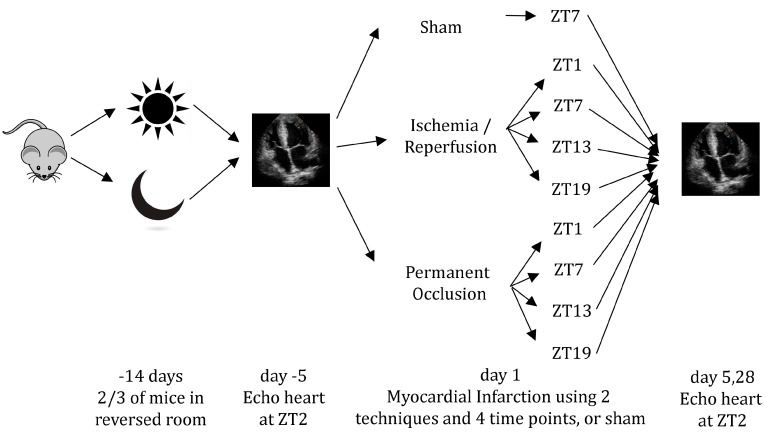
Overview of the procedure. Two-thirds of all mice were placed in rooms with altered light/dark regimes. Five days before the procedure, baseline echocardiography was performed. On Day 1, myocardial infarction (or sham) was induced at four different time-points using two different techniques. At 5 and 28 days, outcome was measured using echocardiography. ZT: Zeitgeber Time (ZT0 = lights on).

Echocardiography was performed under 2% Isoflurane anesthesia 5 days before, 5 days after, and 28 days after the operations using a 13–24 MHz transducer (Vevo 2100 and MS250, Visual Sonics, Toronto, ON, Canada). All measurements were taken at ZT2. During the procedure, heart rate, temperature, and respiration were continuously monitored. Two independent and blinded researchers performed the echo analyses using the manufacturer’s software (version 1.6.0, Visual Sonics, Toronto, ON, Canada). Ejection fraction (EF), end diastolic volume (EDV), and end systolic volume (ESV) values were derived from 3 averaged mid-myocardial long axis recordings in B-mode. Fractional shortening (FS), end diastolic diameter (EDD), and end systolic diameter (ESD) were measured in M-mode. Three short axis recordings from cardiac base to apex were averaged. An overview of the procedures is shown in Figure 5.

### 4.4. Histology

After the last echocardiography, mice were sacrificed by cervical dislocation. The hearts were collected, washed in cold phosphate-buffered saline, weighed (both ventricles), and stored in 4% paraformaldehyde until being embedded in paraffin and sliced in 5 μm long axis sections with 400 μm intervals resulting in between 8–14 total sections. To assess the amount of fibrosis, sections were stained with Picrosirius-red, washed with 0.2n HCl, mounted in entellan, and digitized by scanning. For each heart, a researcher blinded to the origin of the sections, subsequently quantified the amount of fibrosis by dividing the area of collagen by the total left ventricular surface area.

### 4.5. Statistical Analysis

Data are presented as averages ± standard error of mean. Cosinor analysis was used to analyze the presence (or absence) of 24-h rhythmicity in the IR and PL groups. Levene’s test was used to check the equality of variances. When homoscedastic, one- or two-way analysis of variance (ANOVA) was conducted to compare groups. If the data were not homoscedastic, the Kruskal–Wallis test was performed. In analyses with multiple groups, Bonferroni post-hoc analysis was used to compare subjects with controls. Pearson’s chi-squared test was used to compare categorical data. *p*-values < 0.05 were considered statistically significant.

## Figures and Tables

**Table 1:** Cardiac function 28 days after operations.

Function	PL	IR	Sham	PL vs. Sham	IR vs. Sham
EF (%)	31.8 ± 4.0	42.3 ± 1.6	60.4 ± 1.0	*p* < 0.001	*p* = 0.012
FS (%)	19.9 ± 2.6	26.5 ± 1.6	35.7 ± 1.4	*p* < 0.001	*p* = 0.014
EDV (mL)	118.1 ± 18.1	77.3 ± 2.9	64.1 ± 2.8	0.011	1.000

PL: Permanent ligation; IR: Ischemia/reperfusion; EF: Left ventricular ejection fraction; FS: fractional shortening; EDV: end diastolic volume.

## Abbreviations

CVD	Cardiovascular disease
EDD	End Diastolic Diameter
EDV	End Diastolic Volume
ESD	End Systolic DIameter
ESV	End Systolic Volume
EF	Ejection Fraction
FS	Fractional Shortening
MI	Myocardial Infarction
IR	Ischemia/Reperfusion
PL	Permanent Ligation
ZT	Zeitgeber Time
